# The Archaeal Na^+^/Ca^2+^ Exchanger (NCX_Mj) as a Model of Ion Transport for the Superfamily of Ca^2+^/CA Antiporters

**DOI:** 10.3389/fchem.2021.722336

**Published:** 2021-07-30

**Authors:** Daniel Khananshvili

**Affiliations:** Department of Physiology and Pharmacology, Sackler Faculty of Medicine, Tel-Aviv University, Tel-Aviv, Israel

**Keywords:** ion, recognition, transport, NCX, NCKX, NCLX, CAX

## Abstract

The superfamily of Calcium/Cation (Ca^2+^/CA) antiporters extrude Ca^2+^ from the cytosol or subcellular compartments in exchange with Na^+^, K^+^, H^+^, Li^+^, or Mg^2+^ and thereby provide a key mechanism for Ca^2+^ signaling and ion homeostasis in biological systems ranging from bacteria to humans. The structure-dynamic determinants of ion selectivity and transport rates remain unclear, although this is of primary physiological significance. Despite wide variances in the ion selectivity and transport rates, the Ca^2+^/CA proteins share structural motifs, although it remains unclear how the ion recognition/binding is coupled to the ion translocation events. Here, the archaeal Na^+^/Ca^2+^ exchanger (NCX_Mj) is considered as a structure-based model that can help to resolve the ion transport mechanisms by using X-ray, HDX-MS, ATR-FTIR, and computational approaches in conjunction with functional analyses of mutants. Accumulating data reveal that the local backbone dynamics at ion-coordinating residues is characteristically constrained in apo NCX_Mj, which may predefine the affinity and stability of ion-bound species in the ground and transition states. The 3Na^+^ or 1Ca^2+^ binding to respective sites of NCX_Mj rigidify the backbone dynamics at specific segments, where the ion-dependent compression of the ion-permeating four-helix bundle (TM2, TM3, TM7, and TM8) induces the sliding of the two-helix cluster (TM1/TM6) on the protein surface to switch the OF (outward-facing) and IF (inward-facing) conformations. Taking into account the common structural elements shared by Ca^2+^/CAs, NCX_Mj may serve as a model for studying the structure-dynamic and functional determinants of ion-coupled alternating access, transport catalysis, and ion selectivity in Ca^2+^/CA proteins.

## Introduction

Membrane-bound ion transport proteins (channels, transporters, and pumps) can selectively recognize and transport physiologically important cations such as H^+^, Na^+^, K^+^, Ca^2+^, and Mg^2+^ in accordance with the physiological requirements of a given cell type and thereby control the vast majority of biochemical reactions in nearly every living cell (Carafoli, 1987; Berridge et al., 2003; Bers 2008; Forrest et al., 2011; Keller et al., 2014). Ion transporters couple the free energy of the transmembrane electrochemical potential of one solute/ion to the transmembrane movement of another (Bahar et al., 1997; Roux et al., 2010; Forrest et al., 2011; Cheng and Bahar, 2019). The basic paradigm underlying the transporters’ function was put forward more than 5 decades ago by Jardetzky (1966), who described the alternative access of the substrate (ion) binding sites to either one side of the membrane or the other during the transport cycle (Forrest et al., 2011; Drew and Boudker, 2016). According to this phenomenological model, the membrane-bound transporter protein must undergo at least two major conformational states during the transport cycle, designated as the inward-facing (IF) and outward-facing (OF) conformation states.

Today it is clear that transporters undergo numerous conformational changes, although the structure-dynamic segregation and the characterization of functionally important intermediates remain challenging, even for transporters with known structural details (Roux et al., 2010; Lockless, 2015; Deng and Yan, 2016; Drew and Boudker, 2016; Cheng and Bahar, 2019). Structure-dynamic resolution of the underlying ion transport mechanisms may provide new opportunities for selective and effective pharmacological targeting. The relevant information can provide “game-changing” opportunities to advance the most demanding applications in modern biomedicine and technology, although this requires the development of sophisticated multidisciplinary approaches that combine both advanced experimental and computational approaches (Bahar et al., 2010; Roux et al., 2010; Deng and Yan, 2016; Drew and Boudker, 2016; Cheng and Bahar, 2019; Garibsingh and Schlessinger, 2019).

**FIGURE 1:**
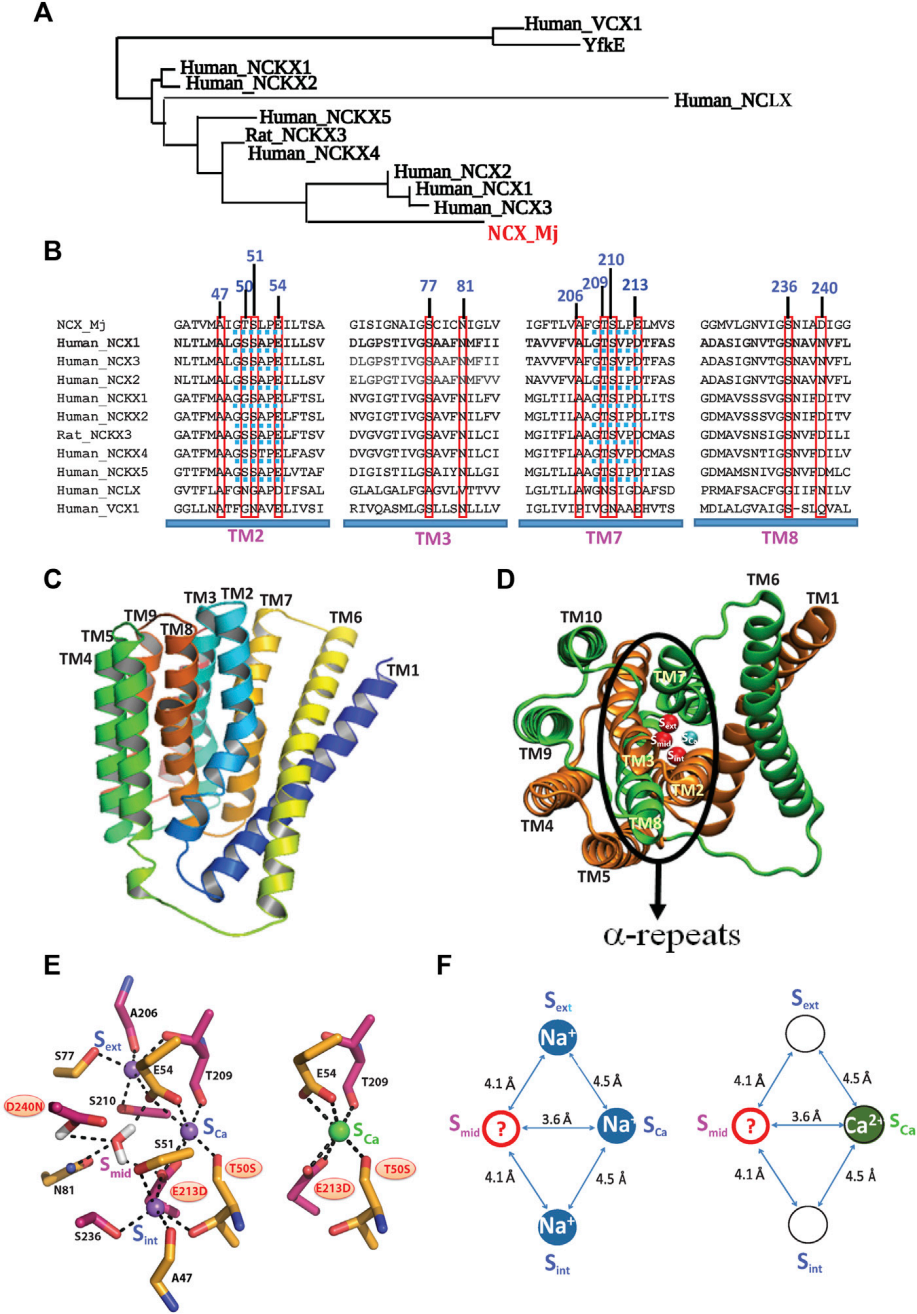
Topological and structural highlights shared by the Ca2+/CA proteins. **(A)** The phylogenetically related gene families, belonging to the superfamily of the Ca^2+^/CA antiporters, share a common topology and structural motifs with inverted twofold symmetry, highly conserved *α*-repeats and ion-coordinating residues. The underlying structure-function relationships are of general interest, since the different gene products can selectively recognize and transport distinct monovalent ions (Na^+^, K^+^, H^+^, and/or Li^+^) in exchange with Ca^2+^. **(B)** Sequence alignments of the NCX, NCKX, NCLX, and CAX proteins (belonging to the superfamily of the Ca^2+^/CA antiporters) contain ion-coordinating residues at four transmembrane helices (TM2, TM3, TM7, and TM8). The red boxes denote the residue overlays of twelve ion-coordinating residues in the Ca^2+^/CA proteins (the numerations of ion coordidinating residues are assigned according to NCX_Mj). Blue dotted lines denote the helix-breaking signiture sequences (similar to GTSPLE) in different Ca^2+^/CA proteins. **(C)** The crystal structure of outward-facing (OF) NCX_Mj (PDB 3V5U) depicts ten transmembrane helices (TM1-TM10). **(D)** Eight helices (TM2-5 and TM7-10) form a tightly packed hub (that is perpendicularly inserted into the membrane), whereas two long and tilted helices (TM1 and TM6) are limply packed in front of a rigid eight-helix core. **(E)** Twelve ion-coordinating residues form four binding sites (S_int_, S_mid_, S_ext_, and S_Ca_). **(F)** NCX_Mj can alternatively bind either 3Na^+^ (at S_int_, S_Ca_, and S_ext_) or 1 Ca^2+^ (S_Ca_). According to this model, the S_Ca_ site binds either the Na^+^ or Ca^2+^ ion, whereas the S_mid_ site can be occupied by a water molecule (through protonated D240), but not by Na^+^ or Ca^2+^.

The aim of the present review article is to summarize our current understanding of the structure-dynamic mechanisms underlying the ion transport mechanisms shared by a huge superfamily of Ca^2+^/Cation (Ca^2+^/CA) exchangers (antiporters). This is achieved by using the structure-based functional model of the archaeal Na^+^/Ca^2+^ exchanger protein derived from *Methanococcus jannaschii* (NCX_Mj) (Figure 1). Even though the structural model of NCX_Mj provides a basis for investigating the general mechanisms underlying ion transport in NCX and similar proteins, certain limitations exist. For example, it is well known that NCX_Mj (like many other archaeal proteins) possesses a very rigid protein structure; therefore, the structure-dynamic features of NCX_Mj should be carefully considered before advocating the general mechanisms of ion transport shared by Ca^2+^/CA proteins. Nevertheless, the structural template of NCX_Mj (Liao et al., 2012; Liao et al., 2016) is instrumental for a structure-based analysis of the ion transport mechanisms in NCX and structurally related proteins (Nishizawa et al., 2013; Waight et al., 2013; Wu et al., 2013; Giladi et al., 2016a; Giladi et al. 2016b; Giladi et al. 2016c; Giladi et al., 2017; Khananshvili 2017).

## Ca^2+^/CA Proteins Share Repetitive Structural Motifs With Inverted Twofold Pseudo-Symmetry

The Ca^2+^/CA superfamily represents a huge group of proteins; it contains at least five gene families (NCX, NCKX, NCLX, CCX, and CAX) (Lytton, 2007; Palty et al., 2010; Emery et al., 2012; Schnetkamp, 2013; Khananshvili, 2020; Assali and Sackler, 2021). The vast majority of Ca^2+^/CAs represent a long-wanted target for practical applications, although this potential intervention is hampered by the lack of structure-dynamic details underlying the ion transport events. The recently discovered high-resolution crystal structures of the archaeal Na^+^/Ca^2+^ exchanger (NCX_Mj) (Liao et al., 2012; Liao et al., 2016) and Ca^2+^/H^+^ exchangers (CAXs) (Nishizawa et al., 2013; Waight et al., 2013; Wu et al., 2013; Lu et al., 2020) provided new insights into the structural organization of the Ca^2+^/CA proteins. In conjunction with this structural information, the highly dedicated MD simulations and advanced biophysical approaches (Almagor et al., 2014; Marinelli et al., 2014; Liao et al., 2016; Giladi et al., 2017; van Dijk et al., 2018) provided new opportunities to resolve the structure-dynamic and functional relationships shared by the Ca^2+^/CA proteins (Giladi et al., 2016a; Giladi et al., 2016b; Giladi et al., 2016c; Khananshvili, 2020).

**FIGURE 2:**
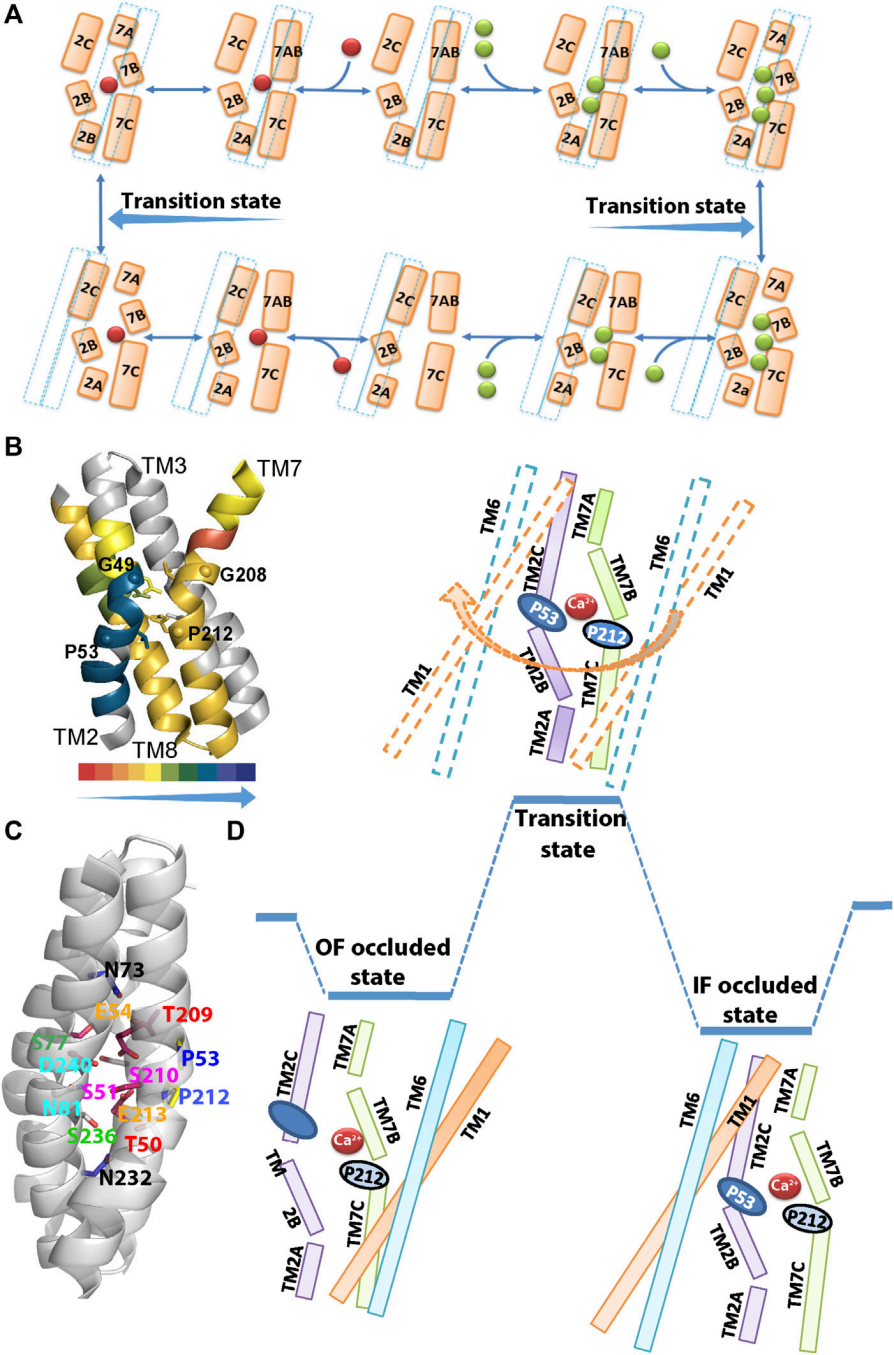
Structural organization of ion binding sites in NCX_Mj. **(A)** The Na^+^/Ca^2+^ exchange cycle involves a separate translocation of the 1Ca^2+^- and 3Na^+^-bound species. Green and red spheres denote the Na^+^ and Ca^2+^ ions, respectively, whereas a dashed line denotes the sliding cluster (TM1/TM6). According to this model, the transition state is situated between the two occluded states, where the sliding event of the TM1/TM6 cluster takes place during the transition state and it is associated with the OF/IF swapping in either direction. **(B)** The backbone dynamics of the four ion-coordinating helices of NCX_Mj are represented by the overlay of the HDX-MS hit maps (Giladi et al., 2017) on the crystal structure of 3Na^+^-bound NCX_Mj (5HXE). The arrow direction indicates the increased rigidity of the backbone dynamics (the color key shows the percentage of deuterium incorporation). Notably, the backbone dynamics of TM2B is much more constrained than the other ion-coordinating segments (TM2C, TM7B, TM7C, and TM8A). Thus, the helix-breaking residues (G49, P53, G208, and P212) belonging to the GTSLPE repeats (allocated at the interface of the TM2B/TM2C and TM7B/TM7C segments) may specifically shape the local backbone dynamics at “catalytic” ion-coordinating residues. **(C)** Single-point mutations of residues allocated at TM2B, TM2C, TM3A, TM7B, TM7C, and TM8A differentially affect the rate-equilibrium relationships of bidirectional ion movements (Giladi et al., 2016a; Giladi et al., 2016b; Giladi et al., 2016c; van Dijk et al., 2018). Inversely matched pair residues (shown in the same colors) differentially affect the ion transport activities, thereby suggesting that the functional asymmetry of inversely located pair residues occurs at the single residue level. **(D)** The occluded and transition states are schematically represented in the case of Ca2+. In the Ca2+ occluded states the hydrophilic gap between the TM2C (P53) and TM7B (P212) segments becomes closer to each other for a hydrophobic patch, although the HDX-MS data reveal that TM2C, TM7B, TM7C, and TM8A, nearby the hydrophobic patch, remain quite flexible upon ion occlusion (Giladi et al., 2017). After the ion occlusion, the hydrophobic patch may undergo further conformational adjustments through the interactions of “catalytic” residues (E54, E213, D240, S51, S77, and T209) with Ca^2+^. As a result, the sliding of the TM1/TM6 cluster can take place to accomplish the OF/IF swapping (the movement of the TM1/TM6 cluster during the transition state is denoted by an arrow). The present considerations may represent the basis for future MD simulations.

In general, the membrane-bound Ca^2+^/CA antiporters extrude Ca^2+^ in exchange with distinct ions (Na^+^, K^+^, H^+^, Li^+^, and Mg^2+^, among others) by utilizing the downhill gradient of counter-cation species such as H^+^, K^+^, or Na^+^ (Blaustein and Lederer, 1999; Philipson and Nicoll, 2000; Lytton, 2007; Emery et al., 2012). Thus, the structure-dynamic model of the archaeal Na^+^/Ca^2+^ exchanger (NCX_Mj) represents a feasible model for studying the ion-transport mechanisms, since twelve ion-coordinating residues (forming four binding sites) are highly conserved among Ca^2+^/CAs, while sharing some common structural motifs (Philipson and Nicoll, 2000; Lytton, 2007; Liao et al., 2012; Schnetkamp, 2013; Marinelli, et al., 2014; Khananshvili, 2020) (Figures 1A,B). Typically, the Ca^2+^/CA proteins contain ten transmembrane helices (TM1–TM10), where two inversely swapped hubs (TM1–TM5 and TM6–TM10) generate an inverted twofold topology with “pseudo-symmetry” (Figures 1A–C). Four transmembrane helices (TM2, TM3, TM7, and TM8) contain highly conserved repeats (*α*_1_ and *α*_2_), where the assembly of the inversely banded TM2/TM3 (*α*_1_) and TM7/TM8 (*α*_2_) segments form an ion passageway with the ion-coordinating residues (Figures 2B,C,D).

## Structure-Functional Assignment of Ion Binding Sites in Ca^2+^/CA Proteins

Prokaryotic and eukaryotic Na^+^/Ca^2+^ exchangers (NCXs) facilitate the transmembrane electrochemical gradient needed to mediate an electrogenic ion exchange with a stoichiometry of 3Na^+^:1Ca^2+^ (Reeves and Hale, 1984; Bers and Ginsburg, 2007; Shlosman et al., 2018). The Na^+^- or Ca^2+^-bound species are separately translocated across the membrane at different stages of the transport cycle (Khananshvili, 1990; Niggli and Lederer, 1991; Hilgemann et al., 1991). High-resolution crystal structures of NCX_Mj capture the outward-facing (OF) conformation with four putative sites, namely, S_ext_, S_mid_, S_int_, and S_Ca_, which are separated from each other by ∼4Å, thereby forming a diamond-shaped configuration (Liao et al., 2012) (Figures 1E,F). Notably, according to the crystal structure, the S_int_ and S_ext_ sites have high selectivity to Na^+^, whereas the S_Ca_ and S_mid_ sites show no preferential selectivity to Na^+^ or Ca^2+^. It was initially proposed that the 3Na^+^ ions occupy the S_ext_, S_mid_, and S_int_ sites, whereas 1Ca^2+^ binds to the S_Ca_ site (Liao et al., 2012). According to this model, NCX_Mj can bind to either the 1Ca^2+^ or 3Na^+^ ions at mutually exclusive sites, meaning that the Na^+^ and Ca^2+^ ions occupy completely different sites at separate steps of the transport cycle.

The follow-up studies with MD simulations and mutational analyses of ion fluxes put forward a revised model for assigning four binding sites in NCX_Mj (Marinelli et al., 2014; Almagor et al., 2014; Giladi et al., 2016a, Giladi et al., 2016b, Giladi et al., 2016c, Giladi et al., 2017, van Dijk et al., 2018; Khananshvil, 2020). According to this model, the 3Na^+^ ions occupy the S_int_, S_ext_, and S_Ca_ sites, whereas the 1Ca^2+^ ion occupies the S_Ca_ site8 (Figures 1E,F). Recent crystallographic studies of NCX_Mj further underscored a unique feature of the S_Ca_ site, which is alternatively occupied by the Na^+^ or Ca^2+^ ion (Liao et al., 2016). The mutational analyses of the ion-exchange activities are in agreement with the revised model of the binding site assignment (Almagor et al., 2014; Marinelli et al., 2014; van Dijk et al., 2018), HDX-MS (Giladi et al., 2016a; Giladi et al., 2016b; Giladi et al., 2016c; Giladi et al., 2017). Despite this progress, the functional status of the S_mid_ site remains unclear. Interestingly, the MD simulations and X-ray data suggest that the S_mid_ site may become occupied by a water molecule through the protonated D240 in either the ion-bound (3Na^+^, 2Na^+^, 2H^+^, or 1Ca^2+^) or apo states (Marinelli et al., 2014; Liao et al., 2016).

High-resolution crystal structures of three H^+^/Ca^2+^ exchanger proteins (all belonging to the CAX family) depict the inward-facing (IF) conformation in the open, semi-open, and occluded states (Nishizawa et al., 2013; Waight et al., 2013; Wu et al., 2013; Lu et al., 2020). According to these structural assignments of the ion-binding sites, the CAX proteins contain the mutually exclusive sites for 1Ca^2+^ (S_Ca_) or 2H^+^ (S_ext_ and S_int_) binding. According to this model, the stoichiometry of the H^+^/Ca^2+^ exchange must be 2H^+^:1Ca^2+^, although experimental evidence for this stoichiometry is absent. Even though the CAXs and NCX share common structural features, the ion selectivity of S_Ca_ might differ in the CAX and NCX_Mj proteins. Namely, according to the crystal structures of CAXs, the S_Ca_ site binds Ca^2+^ (but not H^+^), whereas the S_Ca_ site of NCX_Mj can alternatively bind either Ca^2+^ or Na^+^ (Almagor et al., 2014; Marinelli et al., 2014; Giladi et al., 2016a; Giladi et al., 2016b; Giladi et al., 2016c; van Dijk et al., 2018).

Since the crystal structures of the NCKX and mitochondrial NCLX remain unavailable, the structural template of the NCX_Mj and CAX proteins could be useful for resolving the underlying mechanisms of ion selectivity and transport. Notably, MD simulations have shown that the occupation of four binding sites by 4Na^+^ is thermodynamically forbidden in NCX_Mj (Marinelli et al., 2014; Liao et al., 2016), but not in NCKX (the K^+^-dependent Na^+^/Ca^2+^ exchanger that transports 1Ca^2+^plus 1K^+^ in exchange with 4Na^+^) (Zhekova et al., 2016). This is very intriguing, since ten (out of twelve) ion-coordinating residues (besides T50 and E213) are identical in NCX_Mj and NCKXs (Figure 1B). Thus, NCKX can alternatively bind either 4Na^+^ ions (at S_int_, S_mid_, S_ext_, and S_Ca_) or 1Ca^2+^ (at S_Ca_) plus 1K^+^ (at S_mid_).

Strikingly, nine (out of twelve) ion-coordinating residues differ between the NCLX and NCX_Mj (Figure 1B), where NCX possesses high selectivity for the Na^+^/Ca^2+^ exchange, whereas NCLX can mediate either the Na^+^/Ca^2+^ or Li^+^/Ca^2+^ exchange (Palty et al., 2010; Refaeli et al., 2016; Giladi et al., 2020; Khananshvili, 2020). The replacement of different ion-coordinating residues in NCX_Mj generates a new NCLX_Mj chimera that can generate either Na^+^/Ca^2+^ or Li^+^/Ca^2+^ exchange activities, as a native NCLX does (Refaeli et al., 2016; Giladi et al., 2020). However, the stoichiometry of the Na^+^/Ca^2+^ and Li^+^/Ca^2+^ exchange remains highly uncertain. The inside-positive potential accelerates the NCX_Mj-mediated Na^+^/Ca^2+^ exchange with an electrogenic stoichiometry of 3Na^+^:1Ca^2+^, whereas the NCLX_Mj-mediated Na^+^/Ca^2+^ or Li^+^/Ca^2+^ exchange rates are insensitive to voltage clamp, consistent with the electroneutral (e.g., 2Na^+^:1Ca or 2Li^+^:1Ca^2+^) ion-exchange mode (Giladi et al., 2020; Assali and Sackler, 2021). The HDX-MS and mutational analyses of NCLX_Mj revealed that S_Ca_ binds to Na^+^, Li^+^, or Ca^2+^, whereas the binding of 3Na^+^ (or 3Li^+^) to NCLX_Mj is highly improbable (Refaeli et al., 2016; Giladi et al., 2020). Interestingly, the residues coordinating Ca^2+^ at the S_Ca_ of NCX_Mj (T50, E54, T209, and E213) completely differ from the Ca^2+^-ligating residues of NCLX_Mj (N50, D54, N209, and D213). Namely, two negatively charged residues (E) at the S_Ca_ of NCLX_Mj are replaced by shorter side chain residues (D) and two carbonyl coordinating residues (T) are replaced by rather bulky (N) side chain residues.

### Structure-Dynamic Basis of Ion-Coupled Alternating Access and Transport

In general, the swapping of the IF and OF conformations may occur either in the presence or absence of ligand (ion), whereas an alternating access mechanism takes place differently in distinct groups of “secondary” transporters (Jardetzky, 1966; Forrest et al., 2011; Keller et al., 2014). For example, the action of the antiporter proteins mainly differs from the other groups of secondary transporters (e.g., cotransporters) in that that the ligand (ion) binding to respective sites is required to perform the OF/IF swapping (Forrest et al., 2011; Drew and Boudker, 2016). However, it is unclear how the selective recognition of ions at multiple sites of antiporter proteins is coupled to the ion-induced conformational changes associated with OF/IF swapping. The underlying mechanisms are not trivial to resolve (even for proteins with a known crystal structure), since subtle conformational changes might be involved in the dynamic recognition and coupling of ion transport.

High-resolution crystal structures in the OF state, possessed by NCX_Mj (Liao et al., 2012; Liao et al., 2016), provided the primary clue regarding the major conformational changes underlying the ion-coupled alternating access in the Ca^2+^/CA proteins, otherwise known as the sliding mechanism. According to the originally proposed model, the binding of 3Na^+^ or 1Ca^2+^ to respective sites of NCX_Mj induces the sliding of the tilted two-helix (TM1/TM6) cluster (on the protein surface) in front of the remaining part of the protein (TM2–TM5 and TM7–TM10 hubs). Follow-up discoveries of the CAX structures in the IF state provided further support for the major structural elements involved in the sliding mechanism, thereby suggesting that the Ca^2+^/CA proteins share a common mechanism of ion-coupled alternating access (Nishizawa et al., 2013; Waight et al., 2013; Wu et al., 2013). Despite this progress, the structure-dynamic details underlying the ion-induced movement of the TM1/TM6 bundle remain incompletely understood (Giladi et al., 2016b; Giladi et al., 2017; van Dijk et al., 2018; Khananshvili, 2020).

In the absence of a crystal structure of NCX_Mj in the IF state, the HDX-MS studies provided important complementary information on the structure-dynamic mechanisms underlying the ion-coupled sliding mechanism of NCX_Mj (Giladi et al., 2016b; Giladi et al., 2017). The striking finding is that in both the OF and IF states the TM2B segment is much more constrained (less flexible) than the neighboring TM2C, TM7B, and TM7C segments (Figure 2B). The HDX-MS observations are especially interesting from the perspective of structural evidence indicating that the ion binding/occlusion closes a hydrophilic gap between P53 (TM2C) and P212 (TM7B), thereby yielding a hydrophobic patch (Nishizawa et al., 2013; Giladi et al., 2016b). Even though the occlusion of Na+ or Ca2+ can promote the formation of a hydrophobic patch, the HDX-MS analysis shows that TM2C, TM7B, and TM7C segments remain quite flexible in the ion bound occluded state, though the hydrophobic patch is not stable enough to accomplish the TM1/TM6 sliding.

Extended ion flux analyses of single-point mutations revealed that the side chains of six residues (S51, E54, S77, E213, D240, and T209) are essential for ion transport activities, thereby suggesting that these “catalytic” residues interact with ions in the transition state (Almagor et al., 2014; van Dijk et al., 2018; Iwaki et al., 2020). These data are consistent with the proposal that the TM7B, TM7C, TM2C, and TM8A segments become more stable in front of the “ever-rigid” TM2B upon ion binding/occlusion, where the subsequent interactions of ions with “catalytic” residues in the transition state might drive the TM1/TM6 sliding toward OF/IF swapping (Figure 2). Computer-aided GNM (Gaussian Network Model) analysis of NCX_Mj identified key mechanical sites that potentially act as hinges or anchors for supporting the collective dynamics of the exchanger (van Dijk et al., 2018). These GNM results correlate well with the observed mutational effects on the Na^+^/Ca^2+^ and Ca^2+^/Ca^2+^ exchange rates, thereby identifying functionally important segments, namely, TM2 (G42–S51), TM3 (G76–C80), TM7 (P212–S217), and TM8 (G231–D240), which actively contribute to ion transport and thus, can limit the transport rates. Notably, the single-point mutations of ion-coordinating and non-coordinating residues within these segments have a devastating effect on the transport rates, thus implying that relevant regions play a mechanochemical role in controlling the ion transport rates. Thus, this analysis suggests that single-point mutations within functionally important hinge sites impact the ion-exchange kinetics in NCX_Mj.

In conjunction with GNM analyses, a close examination of collective motions by use of ANM (Anisotropic Network Model) approaches (Bahar et al., 1997; Eyal et al., 2015) have identified the correlated motions of TM1, TM6, and TM7 (block 1) and the strong coupling between TM2, TM3, TM4, TM5, and TM8 (block 2). In conjunction with the HDX-MS and mutational analyses of ion fluxes, the GNM and ANM analyses support the notion that block1 and block2 undergo anticorrelated motions with respect to each other. Thus, they might represent the dynamic features of hinge sites that may control the ion-coupled alternating access in NCX_Mj (van Dijk et al., 2018).

According to the sliding mechanism (Liao et al., 2012), the eight-helix core (TM2-TM5 and TM7-TM10) constitutively remains static (unmoved) during the multiple conformational transitions that take place during the transport cycle. According to this model, the static segments do not undergo any significant conformational changes in the OF/IF swapping, although they provide a protein surface for the TM1/TM6 sliding (Liao et al., 2012; Liao et al., 2016; Nishizawa et al., 2013; Waight et al., 2013; Wu et al., 2013; Lu et al., 2021). In contrast with this assumption, the GNM and ANM analyses revealed that TM4, TM5, TM9, and TM10 of NCX_Mj are quite flexible (van Dijk et al., 2018) and thus, may or may not affect the ion passageway. The structure-dynamic features of these “static” segments as well as their effects on the ion transport activities, must be carefully addressed in future research.

### Structure-Dynamic Hallmarks Associated With Ion Occlusion

The high-resolution crystal structures of NCX_Mj, in conjunction with highly dedicated MD simulations, provided useful information regarding the mechanisms underlying ion recognition and occlusion that take place at the extracellular vestibule in the OF conformation (Marinelli et al., 2014; Liao et al., 2016). These studies have demonstrated that at low concentrations of Na^+^, NCX_Mj adopts the “semi-open” OF conformation, in which two Na^+^ ions occupy the S_int_ and S_Ca_ sites, thereby exhibiting a high affinity occupancy of these sites. At high Na^+^ concentrations, the low affinity binding of the third Na^+^ to the S_ext_ site is associated with a “subtle” backbone bending at the interface of the TM7A and TM7B segments, thereby resulting in the occluded state of 3Na^+^-bound species in the OF conformation. Notably, when Na^+^ binds to the S_ext_ site at high Na^+^ concentrations, the N-terminal part of TM7 bends into two short helices (TM7A and TM7B). According to this mechanism, TM7B occludes all ion-binding sites from the external bulk phase, since the Na^+^ coordination through the backbone carbonyl of A206 and the bulk aromatic ring of F202 prevents ion dissociation from the extracellular vestibule (Liao et al., 2016; Giladi et al., 2017). However, when the S_ext_ site is empty (at low Na^+^ concentrations), the TM7A/TM7B segment is straight (thus, the carbonyl group of A206 and the aromatic ring of F202 are in a pullback position). Moreover, an open gap exists between the bent TM7A/TM7B and the C-terminal half of TM6 when Na^+^ does not occupy the S_ext_ site, whereas the occupation of S_ext_ by Na^+^ generates a hydrophobic patch between two helixes. These ion-induced conformational changes, associated with ion occlusion, are interesting in the context of the sliding mechanism, according to which the ion-coupled sliding of TM1/TM6 on the protein surface promotes the OF/IF switch (Liao et al., 2012, Liao et al., 2016; Marinelli et al., 2014; Giladi et al., 2016b; Giladi et al., 2017).

The ATR-FTIR analysis identified multiple signals for Na^+^ binding to NCX_Mj, which may represent specific steps of ion binding/occlusion at the extracellular vestibule of NCX_Mj (Iwaki et al., 2020). The Na^+^ interactions with respective sites result in Amide I and multi-dentate carboxylate ν_s_ stretches, which represent distinct signals for the high- and low-affinity K_d_ values observed for Na^+^ binding (Iwaki et al., 2020). These observations are consistent with the stepwise binding/occlusion of 3Na^+^ ions at respective sites. Notably, the Amide I signal refers to Na^+^-dependent changes in the secondary structure upon the high-affinity binding of 2Na^+^ ions to the S_int_ and S_Ca_ sites, whereas the changes in the carboxylate IR signal represent the low-affinity binding of the third Na^+^ ion to the S_ext_ site through the ligation of the E213 carboxylate. Collectively, the IR analyses of the apo- and Na^+^-bound species of NCX_Mj reveal the underlying details of 3Na^+^ ion binding/occlusion at the extracellular vestibule in the OF conformation.

HDX-MS (hydrogen-deuterium exchange mass-spectrometry) has been explored to analyze the apo- and ion-bound states of NCX_Mj, with the goal of mapping the backbone dynamic patterns in the OF and IF states (Giladi et al., 2016b; Giladi et al., 2017; Giladi and Khananshvili, 2020). In general, HDX-MS quantifies the exchange rates of backbone amide hydrogen with deuterium in solvent; therefore, more flexible or solvent-exposed regions take up more deuterium than do the rigid segments or the regions that are less exposed to solvent (Demmers et al., 2000; Englander et al., 2003; Chalmers et al., 2011; Konermann et al., 2011; Giladi and Khananshvili, 2020). Thus, HDX-MS is the method of choice for analyzing the local backbone dynamics of ion-occluded states, since relatively small and slow conformational changes in the backbone dynamics can be detected in apo and ligand (ion) bound states (Englander et al., 2003; Chalmers et al., 2011; Konermann et al., 2011).

Notably, the Na^+^ or Ca^2+^ binding to NCX_Mj slightly (but specifically) modifies the backbone dynamics at respective ion-binding sites, although the incremental conformational changes in the OF and IF states are largely predefined by signature landscapes exemplified in the apo-OF and apo-IF protein structures (Giladi et al., 2017). Consistent with the X-ray crystallographic data, the HDX-MS analysis reveals that the Na^+^-dependent bending occurs at TM7AB (202-FTLV-205), where the 3Na^+^ occlusion at the extracellular vestibule may remotely couple the interactions between TM7AB and TM6 (Giladi et al., 2016b; Giladi et al., 2017; Giladi and Khananshvili, 2020). According to the HDX-MS analysis, the bending of TM7AB, upon the occlusion of the 3Na^+^ ions, may affect the hydrophobic interactions between TM6 and TM7 (involving L204), which is consistent with the higher solvent exposure (higher deuterium uptake) associated with the S_ext_ occupation by the third Na^+^ ion, thereby achieving the 3Na^+^ occlusion (Giladi et al., 2017).

## Structure-Dynamic Asymmetry of Bidirectional Access/Permeation in NCX_Mj

Biochemical and kinetic tests revealed that access of ions to the extracellular and cytosolic vestibules of eukaryotic and prokaryotic NCXs is highly asymmetric (Khananshvili et al., 1995, 1996; Almagor et al., 2014; Giladi et al., 2017; van Dijk et al., 2018). This functional asymmetry is especially interesting in the context of *α*1 and *α*2 repeats, since they form an inverted two fold (pseudo)-symmetry for ion passageway in the Ca^2+^/CA proteins. Notably, the repetitive structural elements were generated through the duplication and fusion of genes during evolution; consequently, the functional outcomes of a given protein can be affected in many different ways (Duran and Meiler, 2013; Forrest, 2015; Drew and Boudker, 2016; van Dijk et al., 2018).

In agreement with biochemical studies, the ATR-FTIR analysis revealed the high- and low-affinity K_d_ values for Na^+^ or Ca^2+^ binding to purified NCX_Mj, which may represent the different affinities of ion binding in the OF and IF states (Iwaki et al., 2020). Moreover, the ion-flux assays have shown that the K_m_^Cyt^ values are at least 7–10-times lower than the K_m_^Ext^ values for either Na^+^ or Ca^2+^. Finally, the different K_d_ values measured by ATR-FITR are comparable with the K_m_^Cyt^ and K_m_^Ext^ values measured by the ion-exchange assays (Almagor et al., 2014; Iwaki et al., 2020). Collectively, the available data support the notion that ions have an asymmetric access to the ion-binding pocket at the opposite sides of the membrane under steady-state conditions. Extended mutational analysis of inversely matched pair residues in NCX_Mj revealed that the structure-functional asymmetry occurs at the level of single amino acid residues; this suggests that specific structural elements within the ion passageway differentially control the rate-equilibrium relationships of bidirectional ion movements in NCX_Mj (Giladi et al., 2016a; Giladi et al., 2016b; Giladi et al., 2016c; Giladi et al., 2017; van Dijk et al., 2018).

The HDX-MS analysis of NCX_Mj revealed that ion-passageway entities (involving the TM2, TM3, TM7, and TM8 helices) exhibit characteristic profiles in the local backbone dynamics when they adopt the OF and IF states (Giladi et al., 2016b; Giladi et al., 2017). For example, TM2A and TM8A are more flexible in the IF than in the OF state, whereas the opposite is true for TM7A (Figures 2E–G). Moreover, the OF state is characterized by higher water accessibility at the extracellular than at the cytosolic entry, whereas the opposite is true for the IF state. Thus, pseudo-symmetry-related structural entities at the cytosolic and extracellular entries exhibit reciprocal levels of deuterium uptake (as revealed by HDX-MS) in the IF and OF states. Therefore, they represent hallmark differences in the structure-dynamic preorganization of the OF and IF states prior to ion binding/occlusion. Interestingly, the Na^+^ or Ca^2+^ ion binding to NCX_Mj results in relatively small (but specific) changes in the local backbone dynamics, although the overall conformational asymmetry is largely retained (Giladi et al., 2017). Thus, structure-dynamic preorganization of NCX_Mj underscores the importance of the apo-protein conformational state, which is asymmetric in nature.

### Structure-Functional Hallmarks of Helix-Breaking Signature Sequences

In the Ca^2+^/CA proteins, the inversely oriented helix bends meet each other in the mid-membrane plane, forming helix-breaking structures at the TM2B/TM2C and TM7B/TM7C interfaces in the middle of pore (Figures 1C,D, 2B,C). This structural arrangement contains a highly conserved signature sequence of six residues, which is essential for Ca^2+^ binding/transport (Liao et al., 2012; Liao et al., 2016; Nishizawa et al., 2013; Waight et al., 2013; Wu et al., 2013; Lu et al., 2021) (Figure 1D). Prokaryotic NCX_Mj contains the 49-GTSLPE-54 (TM2B/TM2C) and 208-GTSLPE-213 (TM7B/TM7C) signature repeats, whereas the eukaryotic NCXs contain a modified version of the signature repeats, GSSAPE (*α*_1_) and GTSVPD (*α*_2_), at matching positions (Figure 1D). Notably, the majority of the Ca^2+^/CA proteins possess very similar (but not identical) signature sequences at matching positions, e.g., NCKXs contain similar signature sequences at *α*_1_ (GGSAPE or GSSAPE) and *α*_2_ (GTSIPD or GTSVPD), whereas in the mitochondrial NCLX the signature sequences appear as GNGAPD (*α*_1_) and GNSIGP (*α*_2_) (Figure 1D). Notably, the H^+^/Ca^2+^ exchangers (representing the CAX family of Ca^2+^/CA proteins) possess GNXXE(H) repeats, thereby showing closer similarity to the mitochondrial NCLX.

The present considerations raise the following question: how can the “minute” differences in the signature sequences shape the structure-dynamic features of ion ligation geometry and how can this affect the ion binding affinity, selectivity, and transport rates. Notably, all the relevant structure-dynamic determinants may predefine the physiologically important parameters, such as the ion-binding affinity, the intrinsic asymmetry of bidirectional ion movements, the ion-exchange stoichiometry, and electrogenicity, among many others. Moreover, the relevant structural variations must somehow reconcile with a general mechanism of ion-coupled alternating access shared by Ca (Liao et al., 2012, 2016; Nishizawa et al., 2013; Khananshvili, 2020).

The 49-GTSLPE-54 and 208-GTSLPE-213 repeats contain six ion-coordinating residues (T50, S51, E54, T209, S210, and E213) and four helix-breaking residues (G49, P53, G208, and P212) (Figures 2B–D). The HDX-MS and ion flux analyses of mutants revealed that the folding/unfolding features of the local backbone segments (at the pore center) control the transport rates (Almagor et al., 2014; Giladi et al., 2016a, Giladi et al., 2016b; Giladi et al., 2016c; Giladi et al. 2017; van Dijk et al., 2018). Moreover, the HDX-MS analyses of NCX_Mj revealed that in the apo state the 49-GTSLPE-54 segment is more rigid than its counterpart segment (208-GTSLPE-213); thus, it represents an asymmetrically rigidified structural template for relevant ion-coordinating residues. Interestingly, the binding of the Na^+^ or Ca^2+^ ions specifically (and incrementally) rigidify the backbone dynamics at the respective ion binding sites (Figures 2F,G) (Giladi et al., 2017). Notably, the mutation of the non-coordinating residues (G49, P53, G208, or P212) within 49-GTSLPE-54 and 208-GTSLPE-213 (next to the ion-coordinating carboxylates) have a devastating effect on the transport rates. At this end, it is unclear how the local backbone dynamics at signature sequences control ion ligation in the ground and transition states. The emerging working hypothesis is that characteristic disparities in the local backbone dynamics at signature sequences may control the helix folding/unfolding energies at ion-coordinating residues, consequently, shaping the ion selectivity and transport rates.

## Protonation/Deprotonation Status of Ion-Coordinating Carboxylates in Distinct Orthologs

Notably, the ion-binding pocket of NCX_Mj contains three carboxylates (E54, E213, and D240), whereas the eukaryotic NCXs contain two carboxylates (E54 and D213) at matching positions (Figure 1B). Previous studies with MD simulations and mutational analysis of ion fluxes revealed that D240 of NCX_Mj (exclusively belonging to the S_mid_ site) is protonated, although the deprotonation of D240 is not essential for ion transport activities (Marinelli et al., 2014). In agreement with this, the ATR-FTIR tests have shown that mono-dentate D240 cannot bind to either Na^+^ or Ca^2+^ (Iwaki et al., 2020), thereby suggesting that the S_mid_ site lacks the capacity for Na^+^ or Ca^2+^ binding (at least in the ground state). Accumulating data support a model according to which only two deprotonated carboxylates take part in the 3Na^+^ or 1Ca^2+^ ligation, either in the prokaryotic (E54 and E213) or eukaryotic (E54 and D213) NCX prototypes. According to this model, the 3Na^+^-bound species might carry a positive charge (Z = + 1), whereas the 1Ca^+^-bound species are electroneutral (Z = 0) either in the prokaryotic or eukaryotic NCXs. An important outcome of this model is that the voltage-sensitive translocation of positively charged 3Na^+^-bound species might be a rate-limiting step during the action potential swings in excitable tissues. For example, in cardiomyocytes, the membrane-potential oscillations (from −90 mV to +50 mV and back) might affect the translocation of 3Na^+^-bound species.

Recently performed MD simulations and extended mutational analysis of ion fluxes have shown that the carboxylate residue at the S_mid_ site of NCKX (equivalent to D240 of NCX_Mj) could be in a deprotonated state, thereby allowing the binding of either Na^+^ or K^+^ ions at the S_mid_ site of NCKX (Zhekova et al., 2016; Jalloul et al., 2018). These results seem to be very interesting, since the eukaryotic NCKXs (E54, D213, and D240) and prokaryotic NCX_Mj (E54, E213, and D240) contain three carboxylates within the ion-binding pocket (Figure 1B). However, the striking difference between the eukaryotic NCXs and NCKXs could be that the mono-dentate carboxylate at the S_mid_ site adopts a deprotonated state in NCKX, but not in NCX. This may underscore a fundamental difference between the NCX and NCKX prototypes, although some common electrostatic features can be shared by NCXs and NCKXs as well. More specifically, the 4Na^+^-bound species of NCKX may carry a positive charge (Z = + 1), whereas the 1Ca^+^+1K^+^-bound species might be electroneutral (Z = 0). According to this proposal, the voltage-sensitive translocation of positively charged 4Na^+^-bound species could to be rate limiting during the membrane potential changes in health and disease, thereby showing a certain similarity to the eukaryotic NCXs.

## Allosteric Effects Cannot Explain the Huge Kinetic Variances Among the Prototype Proteins

Even though the prokaryotic and eukaryotic NCXs share a common stoichiometry of ion-exchange (3Na^+^:1Ca^2+^), the turnover rates of the ion-exchange cycle dramatically differ in the eukaryotic NCXs (∼5,000 s^−1^) and NCX_Mj (∼0.5 s^−1^) (Niggli and Lederer, 1991; Hilgemann et al., 1991; Baazov et al., 1999; Almagor et al., 2014). These huge differences in the transport kinetics definitely have a physiological significance for a proper handling of NCX-mediated extrusion of Ca^2+^ from a given cell type, although the structure-dynamic determinants of this fascinating phenomenon remain unresolved (Blaustein and Lederer, 1999; Bers 2008; Khananshvili, 2013; Khananshvili, 2014). The structure-dependent control of kinetic capacities is especially interesting in light of the fact that “only” three (out of twelve) ion-coordinating residues differ among NCXs (T50S, E213D, and D240N) (Figure 1B)–therefore, one may posit that these structural differences may account for kinetic variances (at least partially) among NCXs. However, a systematic replacement of differing ion-coordinating residues in NCX_Mj cannot recapitulate the high turnover rates of those possessed by prokaryotic NCXs (Almagor et al., 2014; van Dijk et al., 2018; Iwaki et al., 2020). These findings are consistent with the notion that the rigid structure of NCX_Mj prohibits an effective ligation of ions in the occlusion and/or transition states, which in turn, may limit the transport rates.

The striking difference in the protein structure of prokaryotic and eukaryotic NCXs is that the cytosolic 5L6 loop (between TM5 and TM6) of eukaryotic NCXs is very long (∼520 residues) and contains the Ca^2+^ binding regulatory domains (CBD1 and CBD2). In contrast, the prokaryotic NCXs have a very short 5L6 loop (12–32 residues) in the absence of regulatory CBD domains (Philipson and Nicolle, 2000; Hilge et al., 2006; Liao et al., 2012). The eukaryotic NCXs undergo an extensive splicing at CBD2 and are expressed in a tissue-specific manner in order to fulfill the functional requirements of a given cell type (Lytton, 2007; Khananshvili, 2016; Khananshvili, 2017; Khananshvili, 2020). Notably, the Ca^2+^ binding to the regulatory CBD domains of eukaryotic NCXs can activate the NCX-mediated transport activities up to 20-fold (Boyman et al., 2011). Thus, at resting levels of cytosolic Ca^2+^, the transport rates of eukaryotic NCX might be at least 10^3^-times faster than those of prokaryotic NCX_Mj. Moreover, the proteolytic shaving of the regulatory CBD domains of the cardiac NCX results in a maximal activation of the transport rate, although the regulatory responses are completely lost (Doering et al., 1996). Interestingly, the elongation of the 5L6 loop of NCX_Mj (by 8–16 residues) results in a 5–10-fold activation of the transport rates (Almagor et al., 2014), thereby suggesting that the 5L6 elongation may facilitate the dynamic features of TM1/TM6 toward OF/IF swapping. Thus, it would be interesting to determine what are the specific structure-dynamic determinants that predefine so high rates in eukaryotic NCXs.

## Concluding Remarks and Perspective

A systematic application of multidisciplinary approaches, which included structural (X-ray crystallography), computational, biophysical (HDX-MS, ATR-FITR), and biochemical techniques, shed light on the ion transport mechanisms operating in the Ca^2+^/CA proteins. High-resolution crystal structures of NCX_Mj (in the OF orientation) and of CAX proteins (in the IF orientation), in the open, semi-open, and occluded conformation states provided a fundamental basis for considering the “sliding mechanism” as a common mechanism for ion-coupled alternating access in Ca^2+^/CA proteins. Follow-up studies with MD simulations and mutational analysis of ion fluxes have established the functional features of individual ion-binding sites and have identified the key residues controlling the ion transport activities in the NCX and NCKX proteins. HDX-MS has identified the ion-induced changes in the local backbone dynamics and thereby provided specific clues for “subtle” conformational changes that accompany the ion-coupled alternating access. ATR-FTIR elucidated the protonation/deprotonation states of ion-coordinating carboxylates, thereby underscoring the charge-carrying features of ion-bound species with different numbers of carboxylates within the ion-binding pocket.

Collectively, the structural and functional data gained during the last years have provided a conceptual framework for studying the dynamic mechanisms by using the template model system of the archaeal NCX_Mj protein. An increasing body of evidence has revealed that the “sliding mechanism” can be considered as a framework model (with specific modifications) for the ion transport mechanisms in the Ca^2+^/CA proteins, although a considerable amount of work is required to resolve subtle conformational changes that seem to be essential for performing the OF/IF swapping during the transport cycle. This is especially true for eukaryotic NCX, NCKX, and NCLX proteins, whose the structural details remain unavailable. This is important since numerous isoform/splice variants of these proteins are expressed in a tissue-specific manner to fulfill cell-specific physiological requirements in a given cell type; From the structure-functional standpoint, the challenge is to determine how the allosteric signals are transferred from distantly located regulatory domains to transport sites and how the output of allosteric signals is modified in distinct variants.

Given the physiological importance of NCX, NCKX, and NCLX proteins in health and disease, a future discovery of mammalian protein structures may provide new opportunities for long-wanted pharmacological targeting of tissue-specific isoform/splice variants. In the long-term, this may have a clinical significance. Taking into account the accumulating data discussed here, the structural template of NCX_Mj can be further explored to better understand the ion transport mechanisms in NCX and similar proteins.

## Abbreviations

ATR-FTIR, Attenuated Total Reflection Fourier-Transform Infrared spectroscopy; CAX, proton-calcium exchanger; CBD1 and CBD2, Ca^2+^ binding regulatory domains of eukaryotic NCXs; HDX-MS, Hydrogen-Deuterium Exchange Mass-Spectrometry; NCKX, K^+^-dependent sodium-calcium exchanger; NCLX, mitochondrial NCX capable of Li^+^ transport; NCX, sodium-calcium exchanger; NCX_Mj, NCX from *Methanococcus jannaschii*; NCX1.1, cardiac isoform/splice variant of NCX1.
